# Unusually Severe Bout in a Patient With a History of Cluster Headache Associated With COVID-19: A Case Report and Review of the Literature

**DOI:** 10.7759/cureus.40781

**Published:** 2023-06-22

**Authors:** Yuzuru Yasuda, Ken Yasuda

**Affiliations:** 1 Neurology, Yasuda Clinic, Kyoto, JPN; 2 Neurology, Kyoto University Graduate School of Medicine, Kyoto, JPN

**Keywords:** headache, angiotensin converting enzyme 2, calcitonin gene-related peptide, incubation period, cluster headache, corona virus disease 2019

## Abstract

Although coronavirus disease 2019 (COVID-19) mainly exhibits respiratory symptoms, neurological symptoms are also reported, with headache being the most common neurological symptom. Headache associated with COVID-19 is widely reported. However, there are few precise case reports concerning headaches in patients with a history of migraine, tension headaches, or cluster headaches associated with COVID-19. Herein, we report a case of a woman with a history of cluster headaches who showed an unusually severe bout 10 days before typical COVID-19 symptoms. Such a case has not been reported until now.

## Introduction

First emerged in Wuhan in December 2019, coronavirus disease 2019 (COVID-19), caused by a novel coronavirus called SARS-CoV-2, has affected millions worldwide, declaring it a pandemic [1]. Although COVID-19 symptoms are mostly respiratory, neurological symptoms have also been reported, with dizziness and headache being the most prominent; anosmia, ageusia, ischemic infarction, and consciousness disturbance have also been reported. Headache associated with COVID-19 has been reported several times [2]; however, it is very complex, and its precise mechanism remains unknown. While a case report concerning a patient with a history of migraine associated with COVID-19 exists in the literature [3], no precise case report about a patient with a history of cluster headaches associated with COVID-19 has been reported. Thus, we present a patient with a history of cluster headaches whose first COVID-19 symptom was an unusually severe bout and the condition's pathogenesis.

## Case presentation

A 45-year-old female visited our clinic with a severe headache. Her medical history revealed that at 25 years old, she began experiencing excruciating pain in the eye, forehead, and cheek on the left side, as well as a runny nose and excessive tearing. The headache usually occurred in spring, 3-4 times per day, often around 2 and 9 am, lasting 2-3 h without treatment. The bout ranged from 1-2 months, and the pain-free period lasted at least three months without preventive treatment, indicating an episodic cluster headache based on the International Classification of Headache Disorders third edition [4].

She described the excruciating pain in the eye, forehead, and cheek on the left side and severe runny nose and tearing as distinct from the usual bout; although the place, time, and duration of the headache were the same as the usual bout, the headache occurred 1-2 times per day, thereby less frequent than the usual bout (3-4 times per day); however, the degree of headache, runny nose, and tearing were more severe than the usual bout and deemed unbearable. The neurological examination and brain MRI showed no abnormality. A complete blood count was unremarkable and C-reactive protein was negative. Subsequently, she was administered verapamil hydrochloride (80 mg) and prednisolone (10 mg) daily. On the bout, rizatriptan benzoate (10 mg) and a nasal drip of sumatriptan succinate (20 mg) were prescribed. However, these treatments were ineffective. Only the subcutaneous injection of sumatriptan succinate (3 mg) was effective.

After six and seven days after her initial bout, her son and husband developed a fever. Polymerase chain reaction (PCR) via nasopharyngeal swabs revealed that they were positive for COVID-19. After 10 days after her initial bout, she also developed a fever and was diagnosed with COVID-19, but anosmia, cough, myalgia, or dyspnea was absent. The fever subsided in a few days. She received a subcutaneous injection of sumatriptan succinate several times during the bout, which subsided 5 weeks after the initial one. After eight months from the initial, no recurrence was reported.

## Discussion

Although the association between headaches and COVID-19 is often reported, the precise mechanism of headaches in COVID-19 remains unknown. In a previous report, approximately 64% of 73 patients associated with COVID-19 presented with headaches, with 51%, 40%, and 26% showing migraine-like, tension-type headache-like, and cough headache (cough headache patients may also have another headache pattern), respectively. Most patients showed headaches on the first day of symptoms [2].

 However, in our patient's case, the headache began 10 days before the fever developed, even though the place, time, and duration of the headache were the same as the usual bout, and the frequency of the headache decreased compared with the usual bout. Conversely, the severity of the headache, runny nose, and tearing were extremely high, suggesting that the degree of the mechanism of the patient's headache was exaggerated compared with the usual bout. A similar finding was obtained in two patients with a previous migraine; a 31-year-old woman with a history of episodic migraine developed a moderate-to-severe headache distinct from her usual migraine. The headache did not sufficiently respond to her usual therapy. One week later, she developed a fever, cough, severe myalgia, dyspnea, and diarrhea, with a positive test for COVID-19 via nasopharyngeal swab PCR. The other patient was a 32-year-old woman with a history of chronic migraine. She developed a severe, intractable headache different from her usual migraine; it was more intense, persistent, and unresponsive to abortive treatment. One week later, she developed typical COVID-19 symptoms, which consisted of low-grade fever, myalgia, nasal congestion, anosmia, and diarrhea, with a positive test for COVID-19 via nasopharyngeal swab PCR [3].

The similarity between the present case and the two abovementioned cases is that the headache developed 7-10 days before the typical COVID-19 symptoms, including fever, cough, myalgia, and diarrhea. Additionally, the severity of the headache was more intense than usual. This finding could provide evidence to clarify the pathogenesis of headaches in a patient with a history of migraine or cluster headaches associated with COVID-19.

The mean incubation period of COVID-19 is reportedly 5.1 days. In a previous study, the onset of COVID-19 symptoms was within 11.5 days in 97.5% of patients [5]. The present case and the abovementioned migraine cases manifested headaches 7-10 days before the typical COVID-19 symptoms, suggesting that the mechanism to trigger headaches starts in these patients' initial stage of the incubation period.

In more than half of patients with cluster headache, the trigeminovascular system is always in an unstable pro-inflammatory condition [6]; consequently, certain stimulation would cause trigeminal activation, calcitonin gene-related peptide (CGRP) release, and trigeminovascular inflammation [7]. For example, seven patients with cluster headaches who had been attack-free for a long time showed a new cluster episode within a few days after COVID-19 vaccinations [8]; thus, COVID-19 vaccinations might have activated a pro-inflammatory state of the trigeminocervical complex.

Angiotensin-converting enzyme 2 (ACE2) is a transmembrane metalloproteinase host receptor for SARS-CoV-2 entry into the cells [9]. ACE is the key enzyme to produce angiotensin (Ang) II, and ACE2 degrades Ang II to produce Ang 1-7, which counteracts the ACE/Ang II/AT1 receptor axis with tissue protection through the Mas receptor [10]. Binding SARS-CoV-2 to the cells downregulates the ACE2/Ang1-7/Mas receptor axis, leading to unbalanced ACE/Ang II/AT1 receptor activity and adverse effects [11]. As the binding affinity of the SARS-CoV-2 spike protein to the human ACE2 receptor on the capillary endothelium increases [12], the blood-brain barrier would be impaired if attacked with COVID-19. By activating the trigeminovascular system, pain-generating neuropeptides such as substance P and CGRP will be released, thereby aggravating migraine or generating headaches similar to migraine [13]. Furthermore, CGRP is one of the key neuropeptides in cluster headaches [14], and its antagonist is effective in treating episodic cluster headaches [15]. Although the precise mechanism of headache in our patient remains unknown, the trigeminovascular system in an unstable condition might be activated by SARS-CoV-2 spike protein in the initial stage of the incubation period, thereby worsening the headache.

## Conclusions

When a patient with a history of migraine or cluster headaches shows unusually severe headaches, clinicians should consider that the headache could be a prodromal stage of COVID-19, and careful follow-up is needed.
